# Aerobic Exercise Improves Type 2 Diabetes Mellitus-Related Cognitive Impairment by Inhibiting JAK2/STAT3 and Enhancing AMPK/SIRT1 Pathways in Mice

**DOI:** 10.1155/2022/6010504

**Published:** 2022-05-05

**Authors:** Lili Lin, Yonghua Wang, Wenli Xu, Chaolu Huang, Jinrong Hu, Xixi Chen, Xinhuang Lv, Yuelin Qin, Xiaoyong Zhao, Haiyan Li

**Affiliations:** ^1^Department of Recovery (Department of Rehabilitation Medicine and Physiotherapy), The First Affiliated Hospital of Wenzhou Medical University, Wenzhou 325000 Zhejiang Province, China; ^2^Department of Physical Education, Wenzhou Medical University, Wenzhou 325800 Zhejiang Province, China; ^3^Department of Anesthesiology, The First Affiliated Hospital of Wenzhou Medical University, Wenzhou, 325000 Zhejiang Province, China; ^4^Department of Neurology, The First Affiliated Hospital of Wenzhou Medical University, Wenzhou 325000 Zhejiang Province, China; ^5^Department of Anesthesiology, Affiliated Hospital of Weifang Medical University, Weifang 261000, China

## Abstract

Type 2 diabetes mellitus (T2DM) is a prevalent risk factor for cognitive impairment. Aerobic exercise can improve T2DM-related cognitive impairment; however, the possible mechanisms remain elusive. Thus, we assessed db/m mice and leptin receptor-deficient (db/db) mice that did or did not perform aerobic exercise (8 m/min, 60 min/day, and 5 days/week for 12 weeks). In this study, cognitive function was significantly impaired in the T2DM mice; aerobic exercise improved cognitive impairment through activating the AMPK/SIRT1 signalling pathway and inhibiting the JAK2/STAT3 signalling pathway in T2DM mice. However, after the application of RO8191 (JAK2 activator) or Compound C (AMPK inhibitor), the positive improvement of the exercise was evidently suppressed. Taken together, our data indicated that long-term aerobic exercise improves type 2 diabetes mellitus-related cognitive impairment by inhibiting JAK2/STAT3 and enhancing AMPK/SIRT1 pathways in mice.

## 1. Introduction

Type 2 diabetes mellitus (T2DM) is becoming an epidemic worldwide, and due to diet or genetic predisposition, an estimated 6.28% of the population experiences this chronic disease [1]. As one of the most serious health problems, T2DM is associated with an increased risk of renal and cardiovascular diseases (CVDs) [2], as well as neurological complications in both the peripheral and central nervous systems [3]. Accumulating evidence demonstrates that T2DM presents significant challenges related to maintaining physical independence and cognitive ability in the context of an ageing population, particularly with regard to cognitive impairment [4]. Individuals with T2DM develop cognitive impairment early and are likely to experience Alzheimer's disease, characterised by cognitive dysfunction [5], which is expected to continue to increase in prevalence over time [1]. Therefore, it is important to alleviate the cognitive impairment caused by T2DM to improve patient independence and quality of life.

Aerobic exercise is increasingly suggested as an effective nonpharmacological strategy to combat cognitive decline in T2DM patients [6]. For instance, Choi et al. identified one of the main proposed mechanisms whereby exercise affects cognitive function by increasing the brain-derived neurotrophic factor (BDNF) [7]. Furthermore, aerobic exercise can also upregulate the expression of synaptic plasticity-associated proteins [8]. These findings imply that exercise may be an effective treatment for diabetes-related cognitive impairment. However, the molecular mechanism by which aerobic exercise improves cognitive function is poorly understood.

Janus kinase 2 (JAK2) is a vital component of nonreceptor tyrosine kinases [9]. Once activated, JAK2 phosphorylates and signal transducer and activator of transcription (STAT) established the downstream target [10]. Increasing evidence suggests that JAK2/STAT3 proteins have important functions in apoptosis and also participate in food intake under leptin-mediated control [11, 12]. Moreover, the JAK2/STAT3 pathway has been found to be related to memory dysfunction [13]. A recent study revealed that mice injected with the JAK inhibitor, AG490, exhibited spatial memory impairment [14]. Based on these data, we speculated that the JAK2/STAT3 pathway is involved in regulating cognitive impairment induced by T2DM; it is unclear whether exercise plays a role in regulating the JAK2/STAT3 pathway.

In addition, an increase in peripheral blood glucose concentration is a typical characteristic of T2DM, which leads to hippocampal energy metabolism disorder [15]. Adenosine monophosphate-activated protein kinase (AMPK), as well as sirtuin 1 (SIRT1), represent two enzymes involved in the regulation of energy metabolism [16]. AMPK, a regulator of body glucose and hippocampal energy balance [17], is widely administered to increase food intake [18] and forms one of the insulin receptor signalling pathways [19]. SIRT1 is one of the most important protective factors for metabolic syndrome. SIRT1 exhibits the ability to significantly extend cell lifespan and enhance neurogenesis by regulating various pathways [20], making it an exciting therapeutic target for neurodegenerative diseases. Thus, the essential role of AMPK/SIRT1 activity in energy metabolism led us to hypothesise that the AMPK/SIRT1 pathway may be related to the exercise-induced regulation in T2DM mice.

Therefore, in the present study, we explored the effect of aerobic exercise on cognitive impairment in T2DM mice and investigated whether the JAK2/STAT3 and AMPK/SIRT1 pathways are involved in the underlying mechanism.

## 2. Materials and Methods

### 2.1. Animals

**Figure 1:**
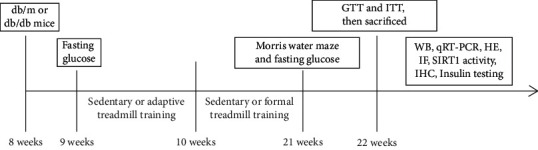
The time duration of animal study.

Each animal experiment was carried out according to the recommendations of the ethics committee guidelines of Wenzhou Medical University (Protocol Number: wxdw 2016-0266). Specific pathogen-free (SPF) male leptin receptor-deficient db/db mice (C57BLKsJ/Nju, 8 weeks) and age-matched male db/m mice (C57BL/KsJNju) were obtained (Strain ID: T002407) from GemPharmatech Co., Ltd. All mice were maintained in conventional laboratory conditions (22°C–25°C, 50%–60% relative humidity), 12 : 12 h light-dark cycle. The time duration of animal study is shown in Figure 1.

### 2.2. Aerobic Exercise Training

At 8 weeks of age, we randomized db/m mice to the control (Con) or exercised (Con+Exe) groups. The db/db mice were randomly assigned to the sedentary (T2DM) and exercised (T2DM+Exe, T2DM+Exe+Vehicle, T2DM+Exe+RO8191, and T2DM+Exe+Compound C) groups. The exercised groups participated in adaptive treadmill training for 1 week (5 m/min, 30 min/day) and thereafter participated in 12 weeks (beginning at 10 weeks of age) of moderate-intensity treadmill training (5 days/week). In the first week of formal training, each animal ran at 8 m/min for 40 min/day; for the remaining weeks, the mice ran at 8 m/min for 1 h/day.

### 2.3. JAK2 Activator and AMPK Inhibitor

The application of JAK2 activator (RO8191) and AMPK inhibitor (Compound C) was conducted as previously reported [21, 22]. In brief, the mice were treated with RO8191 2 mg/kg/day for intraperitoneal injection. Compound C was injected intraperitoneally at 10 mg/kg/day. The T2DM+Exe+Vehicle mice were intraperitoneally inoculated with the solvents without the drug.

### 2.4. Glucose Tolerance Test (GTT) and Insulin Tolerance Test (ITT)

GTT was performed after 12 h fasting by irrigating the stomach with glucose (20%, 2 mg/g body weight). ITT was performed after 6 h fasting by injecting insulin (0.75 mU/g body weight) into the intraperitoneal cavity. Levels of glucose were measured via tail blood before (0 time point) and after 15, 30, 60, and 120 min experiment using an Accu-Check glucometer (Roche, Mannheim, Germany).

### 2.5. Morris Water Maze (MWM) Test

The MWM test was conducted to evaluate the spatial learning and memory abilities of experimental animals. In the 12th week of treadmill training, mice participated in the MWM test. Before starting the MWM test, the mice underwent one-day adaptive training in a circular water pot (height, 50 cm; diameter, 120 cm) containing 22°C white water. Then navigation training (5 consecutive days) and probe test (on day 6) were performed as described previously [23]. The order of the four start positions varied across testing days. A video tracking system (Shanghai Jiliang, China) was used to record.

### 2.6. Fasting Plasma Insulin Testing

The level of fasting plasma insulin was measured by the Mouse Insulin ELISA Kit (Abcam, UK, ab277390).

### 2.7. Western Blotting (WB)

Western blotting was conducted as described previously [6]. The specific information for antibodies and reagents is shown in Table S1. The experiment was repeated three times.

### 2.8. RNA Transcription and Quantitative Real-Time- (qRT-) PCR

Total RNA was isolated from the hippocampus using the RNA simple Total RNA kit (Tiangen, China,), and 0.1 *μ*g RNA was used to prepare cDNA with PrimeScript RT Master Mix (Takara, Dalian, China). For qRT-PCR, 0.3 *μ*l of cDNA was added to 19.7 *μ*l of total PCR reaction mixture, containing SYBR Premix Ex Taq (Roche Diagnostics, Basel, Switzerland). The melting curves of the primers were assessed before use. PCR amplification was performed in 96-well microtiter plates using a 7500 fast PCR machine (Applied Biosystems, Massachusetts, United States). The target gene and house-keeping gene, actin, were run on the same plate as per the protocol. By the *ΔΔ*Ct approach, we measured relative mRNA levels, in which ΔCt = actin Ct − target gene Ct. The qRT-PCR primers are shown in Table S2.

### 2.9. Immunohistochemical (IHC) and Immunofluorescence (IF) Staining

Immunohistochemical and immunofluorescence were performed as outlined previously [9]. Images of IHC were captured under a microscope (×400) and analysed using ImagePro Plus. A fluorescence microscope was used to obtain figures and capture pictures of IF at ×400 magnification. The analysis was conducted using ImageJ. Five digital images of each animal were selected for semi-quantitative analysis. The antibodies are shown in Table S1.

### 2.10. Hematoxylin-Eosin (HE) Staining

The HE was performed by using an HE staining kit (Solorbio, China, G1120). All images were captured at ×400 magnification under a microscope.

### 2.11. SIRT1 Activity

The activity of SIRT1 was measured by tissue Sirtuin 1 activity colorimetric quantitative detection kit (GMS50287.2, GENM2ED SCIENTIFICS INC. USA).

### 2.12. Statistical Analysis

Results are presented in the graphs of mean ± SD. GraphPad 8.0 was adopted for statistical analysis. A normal distribution test was performed using the Shapiro–Wilk test. Comparisons between the two groups were made by Student's *t*-test for statistical significance. Multiple groups were compared using two-way ANOVA or repeated-measures ANOVA and post hoc *t*-test with the Bonferroni correction. *P* < 0.05 represented for statistical significance. The information about degrees of freedom and *F* values is shown in Table S3.

## 3. Results

### 3.1. Aerobic Exercise Improved Blood Glucose and Insulin Level in T2DM Mice

**Figure 2:**
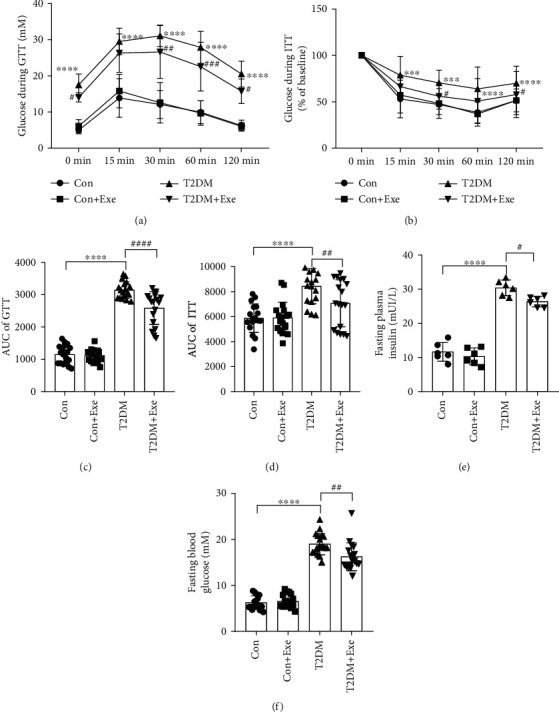
Effect of treadmill exercise in T2DM mice. (a) Glucose during glucose tolerance test (GTT) at age 22 weeks (*n* = 18). (b) Glucose during insulin tolerance test (ITT) (% of baseline) at age 22 weeks (*n* = 18). (c) Area under the curve (AUC) of the GTT (*n* = 18). (d) AUC of the ITT (*n* = 18). (e) Fasting plasma insulin (*n* = 6). (f) Fasting blood glucose (n =18). Data are expressed as mean ± SD. ^∗^*P* < 0.05, significant difference compared with the Con group (^∗∗^*P* < 0.01, ^∗∗∗^*P* < 0.001, ^∗∗∗∗^*P* < 0.001). ^#^*P* < 0.05, which is significantly different from the T2DM group (^##^*P* < 0.01, ^###^*P* < 0.001, ^####^*P* < 0.001).

After 12 weeks of treadmill intervention, T2DM+Exe mice displayed significantly improved in GTT and ITT compared to T2DM mice (*P* < 0.05, Figures 2(a)–2(d)). Compared to the Con group, the levels of plasma insulin and blood glucose were significantly higher in the T2DM group. Exercise improved the insulin and blood glucose levels (*P* < 0.05, Figures 2(e) and 2(f)).

### 3.2. Aerobic Exercise Improved T2DM-Related Cognitive Impairment

**Figure 3:**
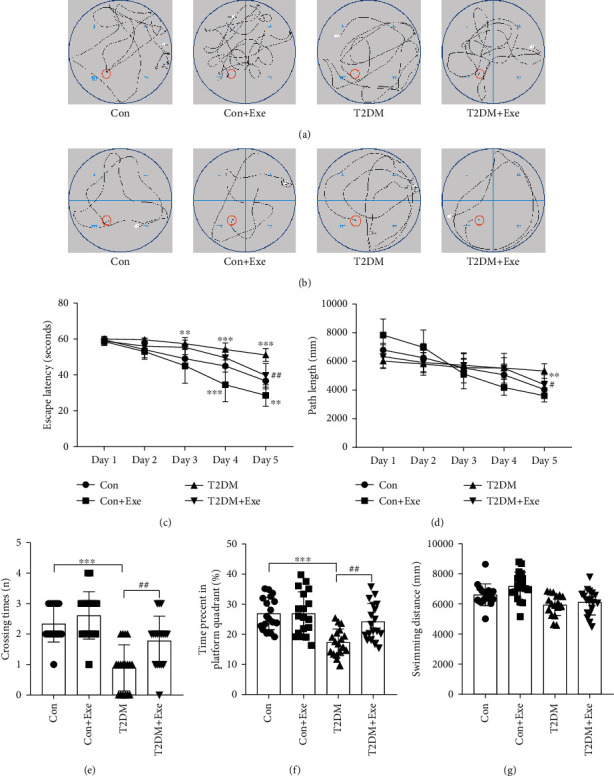
Effect of treadmill exercise on the acquisition of the MWM task. (a) Representative swimming trajectories of mice on the first trial day (*n* = 18). (b) Representative swimming trajectories of mice on the fifth trial day (*n* = 18). (c) Escape latency (*n* = 18). (d) Path length (*n* = 18). (e) Crossing times during the probe test (*n* = 18). (f) Percent of time spent in the platform quadrant during the probe test (*n* = 18). (g) Total swimming distance during the probe test (*n* = 18). Data are expressed as mean ± SD. ^∗^*P* < 0.05, significant difference compared with the Con group (^∗∗^*P* < 0.01, ^∗∗∗^*P* < 0.001). ^#^*P* < 0.05, which is significantly different from the T2DM group (^##^*P* < 0.01).

In this study, we performed the MWM test to investigate the influence of the treadmill on the spatial learning and memory functions of the T2DM mouse. From the representative swimming trajectories of the four groups of mice on the first day of navigation training and latency period, initial differences among the 4 groups were not significant (Figures 3(a) and 3(c)). After training, a significant delay was observed in the T2DM mice, which showed prolonged escape latency and path length compared with the Con mice on the fifth day. The escape latency and path length of the T2DM+Exe mice remarkably decreased compared with the T2DM mice (*P* < 0.05, Figures 3(c) and 3(d)). The representative trajectory of navigation training on the fifth day showed similar results (Figure 3(b)). During the probe test, the number of platform crossings and the time spent in the target quadrant of the T2DM group remarkably decreased in comparison with the Con group. Moreover, the T2DM+Exe mice displayed a higher crossing number and more time staying in the target quadrant compared with the T2DM group (*P* < 0.05, Figures 3(e) and 3(f)). The result of total swimming distances among the four groups was not significant (*P* > 0.05, Figure 3(g)).

### 3.3. Aerobic Exercise Improved Nerve Cell Damage and Protected Synaptic Damage in the Hippocampus Caused by T2DM

**Figure 4:**
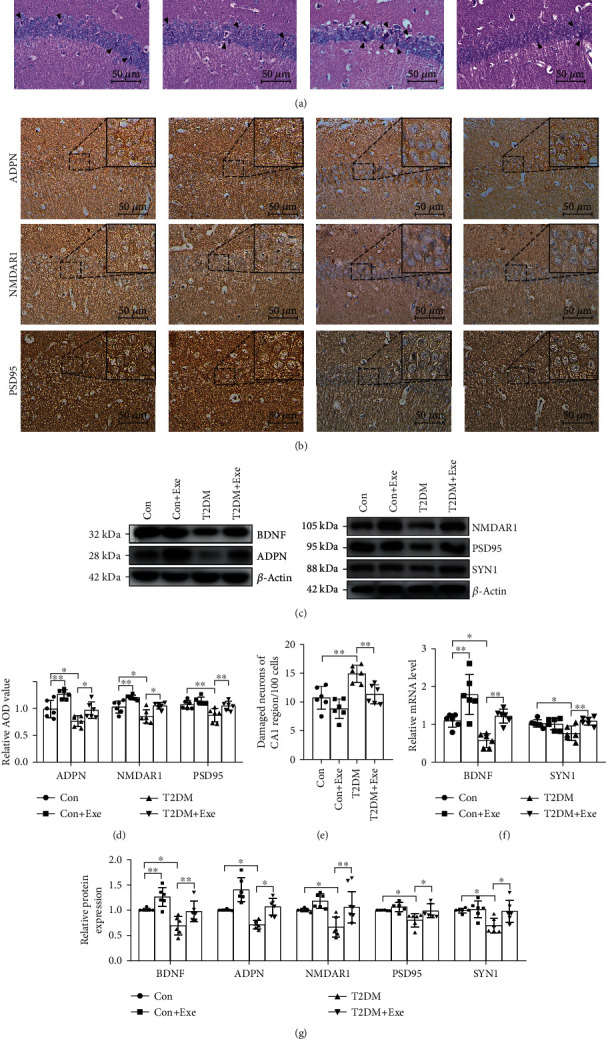
The damage of nerve cells and synaptic in the hippocampus caused by T2DM. (a) Cell histopathological changes in the CA1 region of the hippocampus by HE staining (arrowheads: the damaged neurons). Scale bar: 50 *μ*m (*n* = 6). (b) Immunohistochemical staining in the CA1 region of the hippocampus. Scale bars: 50 *μ*m (*n* = 6). (c) Western blotting (*n* = 6). (d) Immunohistochemical analysis (*n* = 6). (e) The histogram shows the number of damaged neurons of the CA1 region per 100 cells (*n* = 6). (f) The mRNA level (*n* = 6). (g) The histogram shows relative protein levels (*n* = 6). Data are expressed as mean ± SD. ^∗^*P* < 0.05, significant difference (^∗∗^*P* < 0.01).

To observe the damage of hippocampal nerve cells in diabetes, hematoxylin and eosin-stained paraffin sections were used to observe the hippocampal CA1 region. In the Con and Con+Exe groups, most of the neurons were arranged regularly and contained large, round nuclei. In the T2DM group, more nuclei had become pyknotic (contracted and dark, arrowheads in Figure 4(a)) and were surrounded by swollen and broken protrusions. In the T2DM+Exe group, the abnormal structure of the neurons was improved (Figure 4(a)). The number of damaged neurons significantly increased in the T2DM mice and markedly decreased after exercise intervention (*P* < 0.05, Figure 4(e)). In addition, immunohistochemical and Western blotting analyses showed that the positive expression of ADPN, PSD95, and NMDAR1 significantly declined within the T2DM group in comparison with the Con group. This improved in the exercise intervention group (Figures 4(b)–4(d) and 4(g)). Moreover, the protein expression and the mRNA levels of BDNF and SYN1 markedly decreased in the T2DM mice compared with the Con mice and evidently increased after training (*P* < 0.05, Figures 4(c), 4(f), and 4(g)).

### 3.4. Aerobic Exercise Attenuated the JAK2/STAT3 Pathway in T2DM Mice

**Figure 5:**
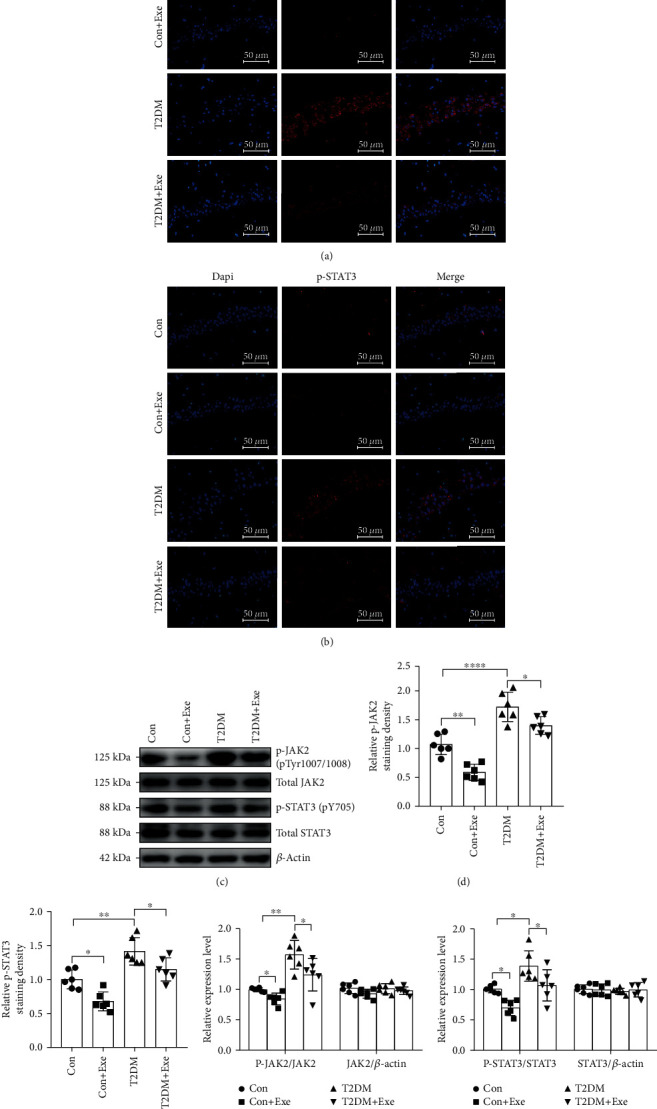
Effect of treadmill exercise on the JAK2/STAT3 signalling pathway in T2DM mice. (a, b) Representative images of p-JAK2 and p-STAT3 (red) in the CA1 region of the hippocampus. Scale bar: 50 *μ*m (*n* = 6). (c) Western blotting (*n* = 6). (d, e) Immunofluorescence analysis (*n* = 6). (f, g) The histogram shows relative protein level (*n* = 6). Data are expressed as mean ± SD. ^∗^*P* < 0.05, significant difference (^∗∗^*P* < 0.01, ^∗∗∗^*P* < 0.001).

We also observed that the hippocampal phospho-JAK2 and phospho-STAT3 levels in the T2DM group were significantly higher than those in the Con mice. With regard to the Con+Exe and T2DM+Exe mice, long-term treadmill significantly decreased phospho-JAK2 and phospho-STAT3 expression (*P* < 0.05, Figures 5(c), 5(f), and 5(g)). As depicted in the immunofluorescence of Figure 5, the same tendency of phospho-JAK2 and phospho-STAT3 levels was observed (*P* < 0.05, Figures 5(a), 5(b), 5(d), and 5(e)).

### 3.5. Aerobic Exercise Activated the AMPK/SIRT1 Pathway in T2DM Mice

**Figure 6:**
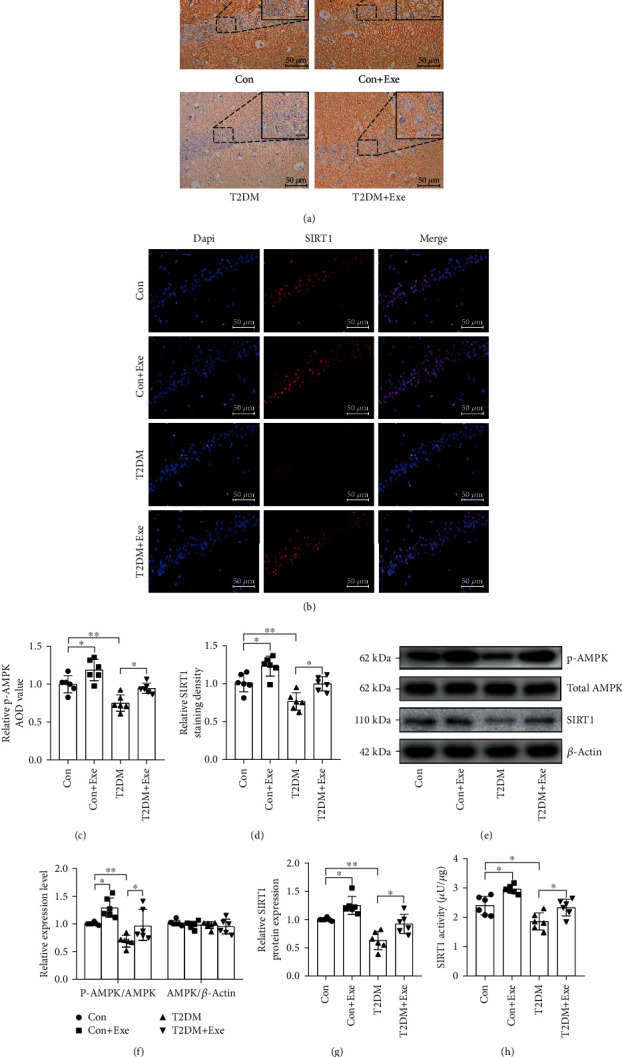
Effect of treadmill exercise on the AMPK/SIRT1 signalling pathway in T2DM mice. (a) Immunohistochemical staining of p-AMPK in the CA1 region of the hippocampus. Scale bars: 50 *μ*m (*n* = 6). (b) Representative images of SIRT1 (red) in the CA1 region of the hippocampus. Scale bar: 50 *μ*m (*n* = 6). (c) Immunohistochemical analysis (*n* = 6). (d) Immunofluorescence analysis (*n* = 6). (e) Western blotting (*n* = 6). (f, g) The histogram shows relative protein levels (*n* = 6). (h) SIRT1 activity (*n* = 6). Data are expressed as mean ± SD. ^∗^*P* < 0.05, significant difference (^∗∗^*P* < 0.01, ^∗∗∗^*P* < 0.001).

To evaluate the effect of exercise on AMPK/SIRT1 pathway, we performed the western blotting. And the SIRT1 and phospho-AMPK expression evidently declined in the T2DM mice compared to the Con mice. In the Con+Exe and T2DM+Exe groups, exercise remarkably elevated SIRT1 and phospho-AMPK expression (*P* < 0.05, Figures 6(e)–6(g)). In addition, the same tendency of phospho-AMPK was observed in immunohistochemical staining, SIRT1 in immunofluorescence staining, and SIRT1 activity (*P* < 0.05, Figures 6(a)–6(d) and 6(h)).

### 3.6. Aerobic Exercise-Induced Improvement in the Learning and Memory Ability of T2DM Mice Was Dependent upon JAK2/STAT3 and AMPK/SIRT1

**Figure 7:**
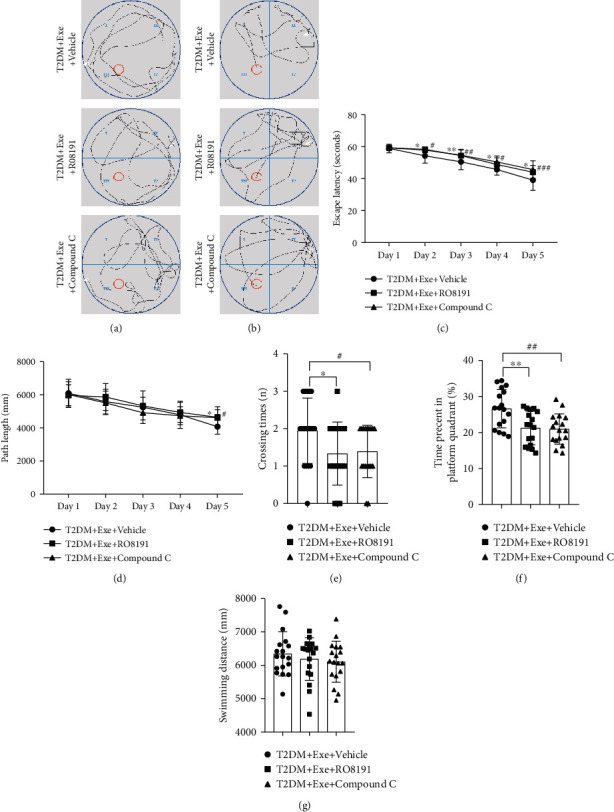
Effect of treadmill exercise on the acquisition of MWM task. (a) Representative swimming trajectories of mice on the first trial day (*n* = 18). (b) Representative swimming trajectories of mice on the fifth trial day (*n* = 18). (c) Escape latency (*n* = 18). (d) Path length (*n* = 18). (e) Crossing times during the probe test (*n* = 18). (f) Percent of time spent in the platform quadrant during the probe test (*n* = 18). (g) Total swimming distance during the probe test (*n* = 18). Data are expressed as mean ± SD. ^∗^*P* < 0.05, significant difference between T2DM+Exe+RO8191 group and T2DM+Exe group (^∗∗^*P* < 0.01, ^∗∗∗^*P* < 0.001). ^#^*P* < 0.05, significant difference between T2DM+Exe+Compound C group and T2DM+Exe group (^##^*P* < 0.01, ^###^*P* < 0.001).

The JAK2 activator RO8191 was used to enhance the activation of JAK2/STAT3. The AMPK inhibitor Compound C was used to inhibit the activation of AMPK/SIRT1 (Supplementary Figure S1). From the representative swimming trajectories on the first day of navigation training and latency period, the difference among the three groups was insignificant (Figures 7(a) and 7(c)). However, escape latency and path length revealed an increase on the fifth day in the T2DM+Exe+RO8191 group compared to the T2DM+Exe+Vehicle group (*P* < 0.05, Figures 7(b)–7(d)). Compared to the T2DM+Exe group, the escape latency and path length of the T2DM+Exe+Compound C group remarkably increased (*P* < 0.05, Figures 7(b)–7(d)). During the probe test, the number of platform crossings and the time spent in the target quadrant of the T2DM+Exe+RO8191 group remarkably decreased in comparison with the T2DM+Exe+Vehicle group (*P* < 0.05, Figures 7(e) and 7(f)). And the T2DM+Exe+Compound C mice displayed a lower crossing number and less time staying in the target quadrant compared with the T2DM+Exe+Vehicle group (*P* < 0.05, Figures 7(e) and 7(f)). There was no significant difference among the three groups for total swimming distances (*P* > 0.05, Figure 7(g)).

### 3.7. Aerobic Exercise-Induced Improvement in Nerve Cell Damage and Protection in Synaptic Damage of T2DM Mice Was Dependent upon JAK2/STAT3 and AMPK/SIRT1

**Figure 8:**
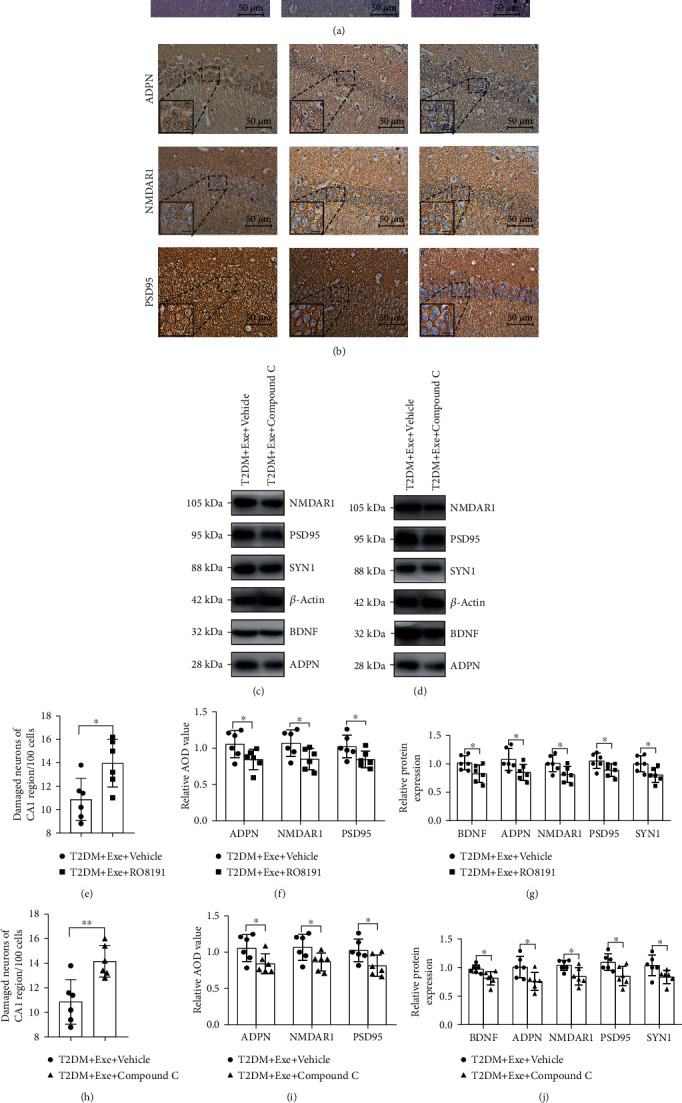
The damage of nerve cells and synaptic in the hippocampus. (a) Cell histopathological changes in the CA1 region of the hippocampus by HE staining (arrowheads: the damaged neurons). Scale bar: 50 *μ*m (*n* = 6). (b) Immunohistochemical staining in the CA1 region of the hippocampus. Scale bars: 50 *μ*m (*n* = 6). (c, d) Western blotting (*n* = 6). (e, h) The histogram shows the number of damaged neurons of the CA1 region per 100 cells (*n* = 6). (f, i) Immunohistochemical analysis (*n* = 6). (g, j) The histogram shows relative protein levels (*n* = 6). Data are expressed as mean ± SD. ^∗^*P* < 0.05, significant difference (^∗∗^*P* < 0.01).

We observed the damage of hippocampal nerve cells in the CA1 region after the application of RO8191 or Compound C. The damaged nerve cells of RO8191-treated mice were significantly more than those of the vehicle mice (*P* < 0.05, Figures 8(a) and 8(e)). In the Compound C-treated mice, it was observed that more nuclei had become pyknotic (contracted and dark, arrowheads in Figure 8(a)) in comparison with the T2DM+Exe+Vehicle group (*P* < 0.05, Figures 8(a) and 8(h)). Further, the positive expression of ADPN, BDNF, SYN1, PSD95, and NMDAR1 significantly declined within the T2DM+Exe+RO8191 group in comparison with the T2DM+Exe+Vehicle group (*P* < 0.05, Figures 8(b), 8(c), 8(f), and 8(g)). Respectively, the positive expression of ADPN, BDNF, SYN1, PSD95, and NMDAR1 evidently decreased after the injection of Compound C (*P* < 0.05, Figures 8(b), 8(d), 8(i), and 8(j)).

## 4. Discussion

In the present study, we found that the 12 weeks of moderate-intensity treadmill training could improve the abnormal glycemia and insulin levels in db/db mice. Additionally, exercise remitted impaired glucose tolerance and insulin resistance. Emerging evidence has demonstrated that cognitive impairments occur in T2DM [24]. We found that cognitive dysfunction related to the function of the hippocampus existed in the T2DM mice, and we revealed that aerobic exercise improved it. In a subsequent investigation, we sought the possible mechanism responsible for the aforementioned phenomenon of recovery of cognitive function via exercise. We observed that phospho-JAK2 and phospho-STAT3 were increased, and the phospho-AMPK/SIRT1 was reduced in T2DM. However, aerobic exercise intervention over a long period improves this situation. Furthermore, JAK2 activator, RO8191, and AMPK inhibitor, Compound C, partly blocked the beneficial effects of aerobic exercise on cognitive function in T2DM mice. These results indicate that JAK2/STAT3 and AMPK/SIRT1 are potential mechanisms by which aerobic exercise improves cognitive decline mediated by T2DM.

Cognitive impairment caused by diabetes can be explained by the loss of synapses caused by insulin resistance (IR). Synaptic dysfunction is the main pathophysiological sign of neurodegenerative diseases [25]. Further, previous studies have indicated that synaptic dysfunction is caused by IR in the brain [26], leading to cognitive decline. However, studies have shown that aerobic exercise could attenuate synaptic loss in models of neurodegenerative disorders, such as Alzheimer's disease [7]. In this study, aerobic exercise mitigated the changes in classical synaptic protein levels observed in T2DM. This is because exercise benefits glucose homeostasis in mice with IR. Glucose homeostasis affects the normal physiological activities of neurons and is able to induce synaptic proteins changes [27]. In this study, our data support that aerobic exercise efficiently improved the impaired cognition caused by diabetes. The expression of three classical synaptic proteins, PSD95, NMDAR1, and SYN1, decreased in the hippocampal CA1 area of diabetic mice, indicating that hippocampal synapse formation in diabetic mice was impaired. The aerobic exercise intervention enhanced hippocampal synaptic formation in T2DM mice. Additionally, we found more abnormal structure neurons in db/db mice, consistent with previous research results [23]. Aberrant insulin modulation in diabetic mice can cause abnormal nerve cell formation. Furthermore, our investigation suggested improvement of neuron shape within hippocampal CA1 region in db/db mice after exercise. Therefore, our research indicates that aerobic exercise has a protective effect on the synapses and neurons in diabetes.

We found that cognitive impairment in T2DM manifests as synaptic disorders and neuronal damage. Recent studies have shown that the JAK2/STAT3 pathway is related to synaptic disorders [28]. JAK2 has a negative impact on synapse formation [29] and insulin sensitivity in diabetes [30]. In addition, the activation of STAT3 has been shown to exacerbate neuronal damage [31]. However, to date, there is a lack of evidence to indicate that aerobic exercise can improve this situation through the JAK2/STAT3 pathway. In our experiments, our data confirmed that phospho-STAT3 and phospho-JAK2 levels were elevated in the mice model of T2DM. In contrast, prolonged aerobic exercise successfully reversed the increased signal of this pathway. Therefore, the data showed that the JAK2/STAT3 pathway is one of the potential mechanisms by which aerobic exercise reduces insulin resistance and protect synapse loss. To verify it, we used RO8191, an activator of JAK2, to activate JAK2/STAT3. We found that the positive effect of exercise on cognitive impairment is reversed after activating JAK2. This indicated that exercise inhibited the JAK2/STAT3 pathway, which is likely one of the mechanisms by which aerobic exercise improves the symptoms of diabetes.

With regard to the AMPK/SIRT1 pathway, evidence has indicated that under pathological conditions, AMPK plays a crucial role in controlling synaptic plasticity and neuronal cell survival [32]. In hippocampal neurons of T2DM mice, the decrease in AMPK phosphorylation is related to the damaged of hippocampal neurons [33], and the activation of phospho-AMPK increases the expression of proteins involved in synaptic plasticity [34]. Moreover, SIRT1 has an important function in regulating neuronal injury [35]. In line with these results, we found that the phospho-AMPK in T2DM was significantly reduced. As confirmed in this study, aerobic exercise upregulated phospho-AMPK and SIRT1 in the hippocampus. Furthermore, we used Compound C, the AMPK inhibitor. Additionally, our data confirmed that the cognitive function improvement by exercise of T2DM mice was partly blocked after suppressing AMPK. Therefore, we believe that aerobic exercise may also prevent synapse formation disorders and improve symptoms of T2DM by activating the AMPK/SIRT1 pathway.

In summary, this study demonstrated that prolonged aerobic exercise can reverse the cognitive decline caused by T2DM, which may be related to the activity of AMPK/SIRT1 and the inhibition of JAK2/STAT3 signalling in T2DM mice. Aerobic exercise may also be used to prevent synapse formation disorders and cognitive decline through enhancing the AMPK/SIRT1 and inhibiting the JAK2/STAT3.

### 4.1. Limitations

Our study discusses the possible mechanism by which aerobic exercise improves T2DM-related cognitive impairment. However, in vitro, there is still no way to achieve aerobic exercise interventions, which are worth investigating in future studies. Meanwhile, we will concentrate on making it used in the clinic trail to verify it.

## Data Availability

Data are available from the corresponding authors upon reasonable request.

## Supplementary Materials

Supplementary 1 The related pathways after application of inhibitors or activators. (A) (D) Western blotting (*n* =6). (B) (C) (E) (F) Histogram shows relative protein levels (*n* =6). Data are expressed as mean ± SD. ^∗^*P* <0.05, significant difference (^∗∗^*P* <0.01).

Supplementary 2 Reagent and antibody.

Supplementary 3 Primers Information.

Supplementary 4 Statistical Information.
