# Supplementary material for: Aerobic Exercise Improves Type 2 Diabetes Mellitus-Related Cognitive Impairment by Inhibiting JAK2/STAT3 and Enhancing AMPK/SIRT1 Pathways in Mice
Source: Dis Markers. 2022 May 5;2022:6010504. doi: 10.1155/2022/6010504 (PMC9107038; doi:10.1155/2022/6010504)
Supplement: Supplementary 1 — The related pathways after application of inhibitors or activators. (A) (D) Western blotting (n =6). (B) (C) (E) (F) Histogram shows relative protein levels (n =6). Data are expressed as mean ± SD. ∗P <0.05, significant difference (∗∗P <0.01). [file 6010504.f1.docx]

**
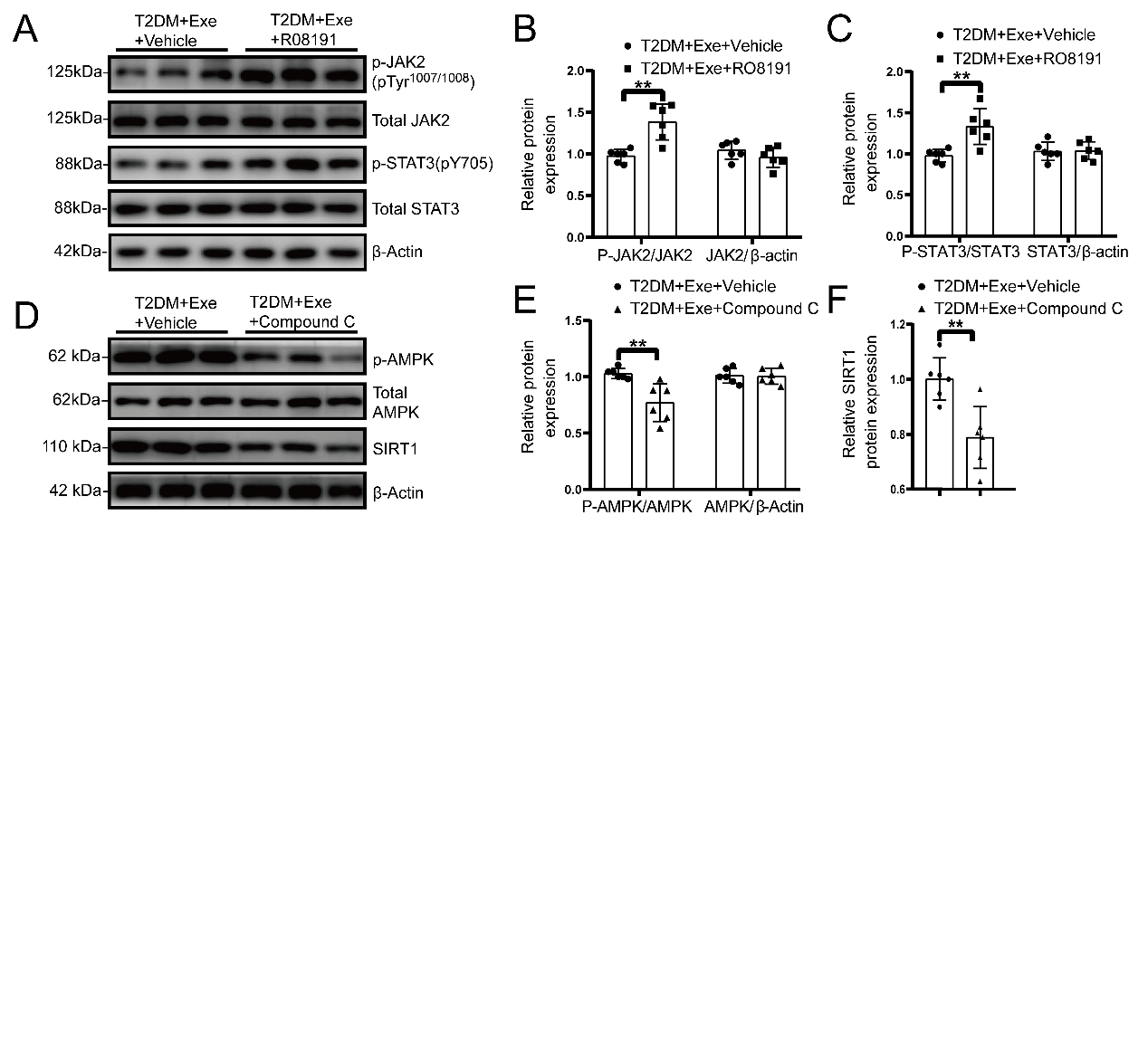
**

**Fig. S1: The Related Pathways after Application of Inhibitors or Activators**

(A) (D) Western blotting (n = 6). (B) (C) (E) (F) Histogram shows relative protein levels (n = 6). Data are expressed as mean ± SD. **P* < 0.05, significant difference (***P* < 0.01).
