# Supplementary material for: Aerobic Exercise Improves Type 2 Diabetes Mellitus-Related Cognitive Impairment by Inhibiting JAK2/STAT3 and Enhancing AMPK/SIRT1 Pathways in Mice
Source: Dis Markers. 2022 May 5;2022:6010504. doi: 10.1155/2022/6010504 (PMC9107038; doi:10.1155/2022/6010504)
Supplement: Supplementary 2 — Reagent and antibody. [file 6010504.f2.docx]

| **Table S1 Reagent and Antibody** | | | |
| --- | --- | --- | --- |
| **Product** | **Application** | **Source** | **Identifier** |
| Antibody |  |  |  |
| Anti-NMDAR1 | WB (1:1000), IHC (1:200) | Abcam, Rabbit, UK | ab109182 |
| Anti-SYN | WB (1:1000) | Abcam, Rabbit, UK | ab254349 |
| Phospho-JAK2 | WB (1:1000), IF (1:200) | Abcam, Rabbit, UK | ab32101 |
| JAK2 | WB (1:1000) | Cell Signaling Technology, Rabbit, USA | #3230 |
| Phospho-STAT3 | WB (1:1000), IF (1:200) | Cell Signaling Technology, Rabbit, USA | #9145 |
| STAT3 | WB (1:1000) | Cell Signaling Technology, Rabbit, USA | #30835 |
| Phospho-AMPKα | WB (1:1000), IHC (1:200) | Cell Signaling Technology, Rabbit, USA | #2535 |
| AMPKα | WB (1:1000) | Cell Signaling Technology, Rabbit, USA | #5831 |
| SIRT1 | WB (1:1000), IF (1:200) | Cell Signaling Technology, Rabbit, USA | #2496 |
| PSD95 | WB (1:1000), IHC (1:200) | Proteintech, Rabbit, USA | 20665-1-AP |
| Adiponectin | WB (1:1000), IHC (1:200) | Proteintech, Rabbit, USA | 21613-1-AP |
| Anti-BDNF | WB (1:1000) | HUABIO, Rabbit, China | ET160642 |
| β-Actin | WB (1:1000) | Proteintech, Rabbit, USA | 20536-1-AP |
| Secondary Antibody | WB (1:5000), IHC (1:200) | Biosharp, Rabbit, China | BL003A |
| Alexa Fluor 594 (red) conjugated secondary antibody | IF (1:200) | Abcam, Rabbit, UK | ab150080 |
| Reagent |  |  |  |
| RO8191 | Activator | GlpBio, USA | HY-W063968 |
| Compound C | Inhibitor | GlpBio, USA | HY-13418A |
| RIPA | WB | Beyotime Biotechnology, China | P0013B |
| PMSF | WB | Beyotime Biotechnology, China | ST506 |
| DTT | WB | Beyotime Biotechnology, China | ST043 |
| Phosphatase inhibitor | WB | Pulilai Gene Technonology, China | P1260 |
| Protein Quantification Kit | WB | BCA, Beyotime Biotechnology, China | P0009 |
| Chemiluminescent substrate | WB | Biosharp, China | BL520A |
| the RNAsimple Total RNA kit | PCR | Tiangen, China | #DP419 |
| PrimeScript RT Master Mix | PCR | Takara, Dalian, China | RR036B |
