# Supplementary material for: Aerobic Exercise Improves Type 2 Diabetes Mellitus-Related Cognitive Impairment by Inhibiting JAK2/STAT3 and Enhancing AMPK/SIRT1 Pathways in Mice
Source: Dis Markers. 2022 May 5;2022:6010504. doi: 10.1155/2022/6010504 (PMC9107038; doi:10.1155/2022/6010504)
Supplement: Supplementary 3 — Primers Information. [file 6010504.f3.docx]

| **Table S2 Primers Information** | | |
| --- | --- | --- |
| **Primers** | **Forward** | **Reverse** |
| BDNF | GCGGCAGATAAAAAGACTGG | TCAGTTGGCCTTTGGATACC |
| SYN1 | AGCTCAACAAATCCCAGTCTCT | CGGATGGTCTCAGCTTTCAC |
| β-actin | AGGCCCCTCTGAACCCTAAG | CCAGAGGCATACAGGGACAAC |
