# Supplementary material for: Aerobic Exercise Improves Type 2 Diabetes Mellitus-Related Cognitive Impairment by Inhibiting JAK2/STAT3 and Enhancing AMPK/SIRT1 Pathways in Mice
Source: Dis Markers. 2022 May 5;2022:6010504. doi: 10.1155/2022/6010504 (PMC9107038; doi:10.1155/2022/6010504)
Supplement: Supplementary 4 — Statistical Information. [file 6010504.f4.docx]

| **Table S3 Statistical Information** | | | | |
| --- | --- | --- | --- | --- |
|  | **Exe:** | **T2DM:** | **Training day/Time:** | **Interaction:** |
| Fig.2 A Glucose during GTT | F (1, 340) = 22.31, P < 0.0001 | F (1, 340) = 853.1, P < 0.0001 | F (4, 340) = 75.59, P < 0.0001 | F (4, 340) = 1.159, P = 0.3.286 |
| Fig.2 B Glucose during ITT | F (1, 340) = 9.97, P = 0.0017 | F (1, 340) = 64.39, P < 0.0001 | F (4, 340) = 120.3, P < 0.0001 | F (4, 340) = 0.8264, P = 0.5091 |
| Fig.2 C AUC of GTT | F (1, 68) = 37.67, P < 0.0001 | F (1, 68) = 1081, P < 0.0001 | / | F (1, 68) = 35.75, P < 0.0001 |
| Fig.2 D AUC of ITT | F (1, 68) = 13.55, P = 0.005 | F (1, 68) = 80.94, P < 0.0001 | / | F (1, 68) = 12.57, P = 0.0007 |
| Fig.2 E Fasting plasma insulin | F (1, 20) = 8.203, P = 0.0009 | F (1, 20) = 345.6, P < 0.0001 | / | F (1, 20) = 2.095, P = 0.1632 |
| Fig.2 F Fasting blood glucose | F (1, 68) = 477.6, P < 0.0001 | F (1, 68) = 5.757, P = 0.0192 | / | F (1, 68) = 8.841, P = 0.0041 |
| Fig.3 C Escape Latency | F (1, 340) = 229.2, P < 0.0001 | F (1, 340) = 84.4, P < 0.0001 | F (4, 340) = 195.4, P < 0.001 | F (4, 340) = 2.566, P = 0.0381 |
| Fig.3 D Path Length | F (1, 340) = 2.368, P = 0.1248 | F (1, 340) = 1.148, P = 0.2848 | F (4, 340) = 91.86, P < 0.0001 | F (4, 340) = 2.896, P = 0.0222 |
| Fig.3 E Crossing times | F (1, 68) = 11.21, P = 0.0013 | F (1, 68) = 42.72, P < 0.0001 | / | F (1, 68) = 3.075, P = 0.084 |
| Fig.3 F Time precent in platform quadrant | F (1, 68) = 6.062, P = 0.0164 | F (1, 68) = 19.66, P < 0.0001 | / | F (1, 68) = 6.121, P = 0.0159 |
| Fig.3 G Swimming distance (mm) | F (1, 68) = 3.883, P = 0.0528 | F (1, 68) = 21.45, P < 0.0001 | / | F (1, 68) = 1.103, P = 0.2973 |
| Fig.4 D Relative ADPN AOD value | F (1, 20) = 26.33, P < 0.0001 | F (1, 20) = 27.25, P < 0.0001 | / | F (1, 20) = 0.1174, P = 0.7354 |
| Fig.4 D Relative NMDA1 AOD value | F (1, 20) = 25.30, P < 0.0001 | F (1, 20) = 13.87, P = 0.0013 | / | F (1, 20) = 0.4177, P = 0.5254 |
| Fig.4 D Relative PSD95 AOD value | F (1, 20) = 12.63, P = 0.0020 | F (1, 20) = 15.01, P = 0.0009 | / | F (1, 20) = 2.615, P = 0.1215 |
| Fig.4 E Damaged neurons of CA1 region | F (1, 20) = 16.54, P = 0.0006 | F (1, 20) = 19.72, P = 0.0003 | / | F (1, 20) = 1.966, P = 0.1762 |
| Fig.4 F mRNA level of BDNF | F (1, 20) = 28.17, P < 0.0001 | F (1, 20) = 19.05, P = 0.0003 | / | F (1, 20) = 0.0287, P = 0.8671 |
| Fig.4 F mRNA level of SYN1 | F (1, 20) = 6.929, P = 0.016 | F (1, 20) = 2.525, P = 0.1278 | / | F (1, 20) = 8.417, P = 0.0088 |
| Fig.4 G Relative ADPN protein expression | F (1, 20) = 34.97, P < 0.0001 | F (1, 20) = 19.98, P = 0.0002 | / | F (1, 20) = 0.0083, P = 0.9282 |
| Fig.4 G Relative BDNF protein expression | F (1, 20) = 15.94, P = 0.0007 | F (1, 20) = 17.49, P = 0.0005 | / | F (1, 20) = 0.1592, P = 0.6942 |
| Fig.4 G Relative NMDAR1 protein expression | F (1, 20) = 17.69, P = 0.0004 | F (1, 20) = 6.485, P = 0.0192 | / | F (1, 20) = 3.348, P = 0.0822 |
| Fig.4 G Relative PSD95 protein expression | F (1, 20) = 8.269, P = 0.0094 | F (1, 20) = 10.19, P = 0.0046 | / | F (1, 20) = 1.756, P = 0.2 |
| Fig.4 G Relative SYN1 protein expression | F (1, 20) = 5.726, P = 0.0266 | F (1, 20) = 6.718, P = 0.0174 | / | F (1, 20) = 4.112, P = 0.0561 |
| Fig.5 D Relative p-JAK2 staining density | F (1, 20) = 27.99, P < 0.0001 | F (1, 20) = 92.6, P < 0.0001 | / | F (1, 20) = 1.112, P = 0.3042 |
| Fig.5 E Relative p-STAT3 staining density | F (1, 20) = 20.47, P = 0.002 | F (1, 20) = 45.52, P < 0.0001 | / | F (1, 20) = 0.1712, P = 0.6834 |
| Fig.5 F Relative expression level (p-JAK2/JAK2) | F (1, 20) =10.3, P = 0.0044 | F (1, 20) = 41.18, P < 0.0001 | / | F (1, 20) = 1.377, P = 0.2544 |
| Fig.5 G Relative expression level (p-STAT3/STAT3) | F (1, 20) = 18.09, P = 0.0004 | F (1, 20) = 18.7, P = 0.0003 | / | F (1, 20) = 0.0012, P = 0.9725 |
| Fig.6 C Relative p-AMPK AOD value | F (1, 20) = 17.91, P = 0.0004 | F (1, 20) = 29.45, P < 0.0001 | / | F (1, 20) = 0.0059, P = 0.9344 |
| Fig.6 D Relative SIRT1 staining density | F (1, 20) = 22.66, P = 0.0001 | F (1, 20) = 25.86, P < 0.0001 | / | F (1, 20) = 0.0029 P = 0.8661 |
| Fig.6 F Relative expression level (p-AMPK/AMPK) | F (1, 20) = 38.38, P < 0.0001 | F (1, 20) = 9.878, P = 0.00051 | / | F (1, 20) = 1.29, P = 0.2695 |
| Fig.6 G Relative SIRT1 protein expression | F (1, 20) = 22.21, P = 0.0001 | F (1, 20) = 36.17, P < 0.0001 | / | F (1, 20) = 0.1339, P = 0.7183 |
| Fig.6 H SIRT1 Activity | F (1, 20) = 21.95, P = 0.0001 | F (1, 20) = 29.58, P < 0.0001 | / | F (1, 20) = 0.1435, P = 0.7088 |
|  | **Vehicle-RO8191** | | **Vehicle-Compound C** | |
| Fig.7 C Escape Latency | F (4, 170) = 115.7, P < 0.0001 (Training day)  F (1, 170) = 31.53, P < 0.0001 (RO8191)  F (4, 170) = 1.743, P = 0.1428 (Interaction) | | F (4, 170) = 17.86, P < 0.0001  (Training day)  F (1, 170) = 6561, P < 0.0001  (RO8191)  F (4, 170) = 17.02, P < 0.0001  (Interaction) | |
| Fig.7 D Path Length | F (4, 170) = 3.149, P = 0.0778  (Training day)  F (1, 170) = 30.86, P < 0.0001  (RO8191)  F (4, 170) = 1.129, P = 0.3446  (Interaction) | | F (4, 170) = 32.64, P < 0.0001  (Training day)  F (1, 170) = 16.28, P = 0.0995  (Compound C)  F (4, 170) = 1.935, P = 0.1068  (Interaction) | |
| Fig.7 E Crossing times | t = 2.14, P = 0.0396 | | t = 2.110, P = 0.0423 | |
| Fig.7 F Time precent in platform quadrant | t = 3.134, P = 0.0037 | | t = 3.453, P = 0.0016 | |
| Fig.7 G Swimming distance (mm) | t = 0.7203, P = 0.4763 | | t = 1.114, P = 0.2733 | |
| Fig.8 E/H Damaged neurons of CA1 region | t = 2.793, P = 0.019 | | t = 3.647, P = 0.0045 | |
| Fig.8 F/I Relative ADPN AOD value | t = 2.232, P = 0.049 | | t = 2.319, P = 0.0429 | |
| Fig.8 F/I Relative NMDA1 AOD value | t = 2.285, P = 0.0454 | | t = 2.277, P = 0.046 | |
| Fig.8 F/I Relative PSD95 AOD value | t = 2.241, P = 0.048 | | t = 2.392, P = 0.0378 | |
| Fig.8 G/J Relative BDNF protein expression | t = 2.372, P = 0.0392 | | t = 2.799, P = 0.0188 | |
| Fig.8 G/J Relative ADPN protein expression | t = 2.320, P = 0.0428 | | t = 2.498, P = 0.0315 | |
| Fig.8 G/J Relative NMDAR1 protein expression | t = 2.401, P = 0.0373 | | t = 2.685, P = 0.0229 | |
| Fig.8 G/J Relative PSD95 protein expression | t = 2.259, P = 0.0457 | | t = 2.727, P = 0.0213 | |
| Fig.8 G/J Relative SYN1 protein expression | t = 2.515, P = 0.0307 | | t = 2.291, P = 0.045 | |
| Fig.S1 B Relative expression level (p-JAK2/JAK2) | t = 3.849, P = 0.0032 | | / | |
| Fig.S1 C Relative expression level (p-STAT3/STAT3) | t = 3.766, P = 0.0037 | | / | |
| Fig. S1 E Relative expression level (p-AMPK/AMPK) | / | | t = 3.641, P = 0.0045 | |
| Fig. S1 F Relative SIRT1 protein expression | / | | t = 3.838, P = 0.0033 | |
